# The Injection Molding of Biodegradable Polydioxanone—A Study of the Dependence of the Structural and Mechanical Properties on Thermal Processing Conditions

**DOI:** 10.3390/polym14245528

**Published:** 2022-12-16

**Authors:** Jakub Erben, Katerina Blatonova, Tomas Kalous, Lukas Capek, Lubos Behalek, Martin Boruvka, Jiri Chvojka

**Affiliations:** 1 Department of Nonwovens and Nanofibrous Materials, Faculty of Textile, Technical University of Liberec, 461 17 Liberec, Czech Republic; 2 Department of Technologies and Structures, Faculty of Textile, Technical University of Liberec, 461 17 Liberec, Czech Republic; 3 Department of Engineering Technology, Faculty of Mechanical Engineering, Technical University of Liberec, 461 17 Liberec, Czech Republic

**Keywords:** injection molding, polydioxanone, degradable polymers, biopolymers, mechanical properties, medical devices

## Abstract

Recent years have observed a significant increase in the use of degradable materials in medicine due to their minimal impact on the patient and broad range of applicability. The biodegradable polymer Polydioxanone (PDO) provides a good example of the use of such one polymer that can represent the aforementioned medical materials in the field of medicine, due to its high level of biocompatibility and interesting mechanical properties. PDO is used to produce absorbable medical devices such as sutures and stents, and is also suitable for the fabrication of certain orthopedic implants. Polydioxanone can be processed using the injection molding method due to its thermoplastic nature; this method allows for the precise and easily-controllable production of medical materials without the need for toxic additives. A number of small commercial polymer implants have recently been introduced onto the market based on this processing method. It is important to note that, to date, no relevant information on the molding of PDO is available either for the scientific or the general public, and no study has been published that describes the potential of the injection molding of PDO. Hence, we present our research on the basic technological and material parameters that allow for the processing of PDO using the laboratory microinjection molding method. In addition to determining the basic parameters of the process, the research also focused on the study of the structural and mechanical properties of samples based on the thermal conditions during processing. A technological frame work was successfully determined for the processing of PDO via the microinjection molding approach that allows for the production of samples with the required homogeneity, shape stability and surface quality in a laboratory scale. The research revealed that PDO is a polymer with a major share of crystalline phases, and that it is sensitive to the annealing temperature profile in the mold, which has the potential to impact the final crystalline structure, the fracture morphology and the mechanical properties.

## 1. Introduction

The field of regenerative medicine has, in recent years, expanded to include the application of resorbable implants; hence, the necessity for the use of biodegradable polymers has increased significantly, especially with respect to synthetic thermoplastics. Such materials exhibit good rheological properties, and they can be processed using standard melt shaping techniques [1]. Polymers based on this group of materials provide very good biocompatibility and inter-batch stability and are becoming increasingly affordable, which makes them attractive with respect to a wide range of medical applications [2].

One of the polymers included in this group of materials, i.e., Polydioxanone (PDO), is used in the field of medicine in the form of surgical sutures [3] and resorbable stents [4]. Thanks to its medical harmlessness and declared unique properties, PDO has been approved by the FDA for use in the field of medical implants. It is a thermoplastic, semi-crystalline polymer from the poly-ether-esters group and it exhibits high levels of biocompatibility, histoconductivity and resorbability. The glass transition temperature is approx. −10 °C and the melting point is 110 °C. It is a relatively tough polymer with a very good shape memory, and attains a crystallinity level of up to 60%. The Young’s modulus value is 1.5 GPa at a relative elongation of 30% (as is the case for surgical monofilaments). The material degrades principally via hydrolytic processes at the time of a 50% loss of the initial breaking strength after a 5-week period [5]. The full resorption state is attained after 6 months via decomposition on the basic metabolites excreted by the patient [6]. The main advantage of PDO (together with rapid resorption) concerns its glass transition temperature of below 0 °C, which allows it to retain a degree of toughness that acts to prevent the breakage of the material, which occurs when using polylactic acid (PLA) and polylactic-co-glycolic acid (PLGA). In addition to the above-mentioned surgical monofilaments and stents, PDO implants are available in the form of pins and small plates for orthopedic fixation [7]. PDO has also been used experimentally in the field of regenerative medicine for the creation of tissue scaffolds [8,9] for drug delivery systems [10,11].

Injection molding comprises the most stable and widely-used method for the production of high-quality, shape-accurate medical devices based on PDO. It provides a highly-efficient method for the production of very precise models, of which the molded part is often the final product [12]. Injection molding is a thermally strictly-controlled process with a range of thermal profiles that significantly affect the final properties of the product. Other important factors concern the properties of the thermoplastic material used, especially its rheological properties and thermal stability [13].

Injection molding is becoming an increasingly popular method for the processing of biodegradable materials. Polylactic acid (PLA) [14,15], thermoplastic starch (TPS) [16], polyhydroxyalkanoates (PHA) [17,18] and polycaprolactone (PCL) [19,20] are used for a wide range of purposes. One of the most important aspects in terms of the processing of biodegradable plastics concerns their susceptibility to degradation as a result of thermal strain, especially when subjected to shear forces. This shortcoming manifests itself principally via a decrease in the molecular weight [21]. The elimination of this negative aspect requires the application of minimal molding temperatures that are sufficient to ensure the suitable rheological properties of the melt. This results in the availability of a relatively narrow technological “window” for the processing of the afore-mentioned group of polymers, which complicates their evaluation in terms of injection molding [22]. The use of various non-isothermal injection mold cooling profiles revealed that it is possible to influence the crystalline structure of polymers with a higher portion of crystalline phases such as PLA or PCL, which results in a major change in the mechanical properties of the final product [23]. In the case of the production of medical implants, injection molding is able (under specific conditions) to work as a sterilizing process with regard to biodegradable polymers [24].

PDO appears to be a good material in terms of its processability using the injection molding method. The complex viscosity of the PDO melt attains a slightly higher value than that of the usually processed PLA. PDO exhibits a higher level of crystallinity than any of the other biodegradable thermoplastic polymers, which provides the potential to significantly influence the mechanical properties of the material related to the change in the quality of the crystalline structure based on the non-isothermal cooling profile [1]. This behavior is expanded upon in publications that describe the ability of PDO to crystallize into objects in a size range between small lamellar structures and large crystallized grains [25]. The predisposition of PDO to degradation during thermal processing has already been demonstrated, particularly in the case of higher molecular weights exposed to longer periods of stress than the usual injection molding time [26].

No paper has been published to date that addresses the investigation of issues surrounding injection molding technology for the processing of PDO. That is not to say that such technology has ever been used for PDO; indeed, it is highly probable that this technology has been used for the manufacture of the afore-mentioned orthopedic pins and small plates. However, the production process was purely industrial without any reference to the scientific aspects. This study addressed the research of the potential for the use of injection molding for the processing of PDO. The used laboratory injection equipment suffers from some technological limitations, and did not permit a detailed description of all technological variables occurring on an industrial scale, which was not the aim of this study. The main objective was to determine the basic technological-processing conditions in tandem with the testing of the material and mechanical properties of models with respect to the use of the thermal profile during processing. Furthermore, this study aimed to prove the validity of the PDO melt crystallization mechanisms described in the publications [1,25,26,27] mentioned above, but directly in the injection molding technology and also to describe their influence on the mechanical properties. The study also included an assessment of the final process parameters and the impact on changes in the molar mass and toxicity. The sensitivity of PDO to the thermal profile, especially during the cooling of the mold, provided an efficient tool for the production of materials with specific mechanical properties tailored for the final application.

## 2. Materials and Methods

### 2.1. Materials

The polydioxanone (PDO) biodegradable (human use approved) thermoplastic polymer—Monosorb^®^, (Samyang Biopharm, Seongnam, South Korea) was used for injection molding purposes. The polymer was obtained in the form of filaments with diameters of 0.65 mm, which were cut into granules with lengths of 1–3 mm.

### 2.2. Material Properties

The thermal stability of the PDO was tested via thermogravimetric analysis (TGA) using a thermogravimetric analyser—Q500 (TA Instruments, USA). The experiment was performed in a synthetic oxygen atmosphere at a flowrate of 60 mL min^−1^ in the temperature range from 25 °C to 750 °C and at a heating rate of 10 °C min^−1^. The thermal stability limit was determined as the temperature at which the weight of the sample decreased by 0.3 wt%.

The crystallinity and transition temperatures of the material were evaluated using differential scanning calorimetry (DSC). A calorimeter—DSC 1/700 (Mettler Toledo, Urdorf, Switzerland) was used for the measurements following calibration according to the indium and zinc standards. The measurements were taken on 10 ± 0.5 mg of the samples in a N_2_ atmosphere with a heating rate of 10 °C min^−1^ from −50 °C to 150 °C. The samples were prepared from cross-sections of the injection molding parts on a rotating microtome—Leica RM255 (Leica Biosystem, Nussloch, Germany). The analysis was performed with a N_2_ flow rate of 50 mL min^−1^. The degree of crystallinity in % (Xc) was taken from the heating run and calculated as:

Xc = [(ΔHm − ΔHc)/ΔHm°] × 100

where ΔHm is the enthalpy of melting and ΔHc is the exothermal crystallization determined from the DSC, and ΔHm° is the enthalpy of melting for the 100% crystalline polymer 141.18 J g^−1^ for PDO [28]. The presented curves represent the averages of two separate measurements in all cases.

The melt flow volume rate (MVR), which describes the flow properties of plastic materials, was measured using a melt flow tester (Ceast, Pianezza, Italy) in the range from 110 °C to 160 °C according to the TGA curve. Thus the temperature dependence of the MVR (cm^3^ 10min^−1^) for the PDO granulate was compiled. A total of 10 measurements were performed for each temperature, which was followed by the calculation of the average values.

Potential shifts in the molar mass following the injection molding process were assessed by means of gel permeation chromatography (GPC) using a high-pressure liquid chromatograph—Ultimate 3000 (Dionex, Sunnyvale, CA, USA). A gel column—PFG Micro 300A (Polymer Standards Service, Mainz, Germany) and a detector—Varian LC-385 ELSD (Agilent Technologies, Santa Clara, CA, USA) were employed. The PDO was dissolved and eluted in 1,1,1,3,3,3-hexafluoro-isopropanol (HFIP) so as to obtain a final concentration of 2 mg/mL. The injection volume of the dissolved polymers was set at 15 μL. The number and weight average molar masses were calculated using polymethyl methacrylate standards for calibration, described in more detail in the supplementary information Figure S1.

### 2.3. Injection Molding

**Figure 1:**
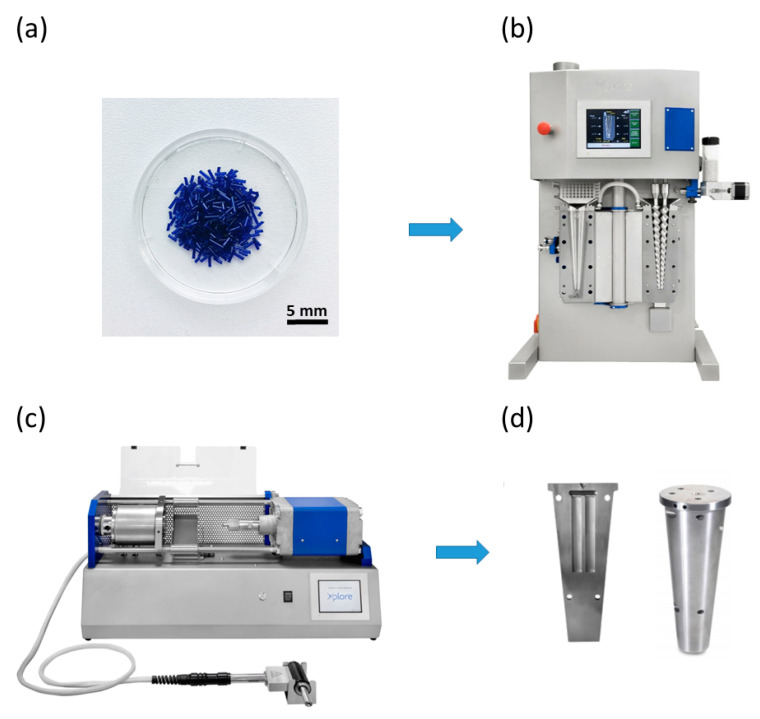
Scheme of the micro injection molding processing: PDO granulate−(**a**), twin-screw compounder−(**b**), micro injection machine−(**c**) and injection mold−(**d**).

The PDO granulate was processed using microinjection molding technology (Figure 1). The granulate was first melted and mixed using a co-rotating twin-screw micro compounder—MC15 (Xplore, Sittard, Netherlands) at a melt temperature of 150 °C, an average holding time of 90 s and a screw speed of 100 rpm. Subsequently, the melt was transferred using a heated, removable transfer unit in a micro injection molder—IM12 (Xplore, Sittard, Netherlands) heated to 160 °C and injected into the mold at two different mold temperatures, i.e., 25 °C and 65 °C (the exact conditions are listed in Table 1). Samples from both molds were removed after 240 s during which time they were allowed to crystallize in the mold. The mechanical test specimens: tensile test dog bone—1BA and flexural/impact test bar (80 mm × 10 mm × 4 mm) corresponded to international standards EN ISO 527-2 and EN ISO 178.

### 2.4. Morphology Characterisation

The structure of the samples was assessed via scanning electron microscopy (SEM) using a VEGA3 SBU—EasyProbe microscope (Tescan, Brno, Czech Republic) equipped with a Tungsten heated cathode as the electron gun and an Everhart-Thornley-type (YAG Crystal) integrated secondary electron detector at an accelerating voltage of 10 kV, an aperture of 20 μm and a working distance of 10–14 mm. The dry samples were coated with a 5 nm-thick gold layer prior to the analysis.

Polarized-light Optical Microscopy (POM) was employed to observe the internal spherulitic structure. Thin sections of a thickness of 10 μm were used for the recording of images in the transmission mode using a polarized-light optical microscope—DSX510 (Olympus, Hamburg, Germany). The thin sections were prepared from cross-sections of the injection molded parts (always from the same location in the center of the shot—flexural test bars with dimensions 80 mm × 10 mm × 4 mm) on a rotary microtome—RM2255 (Leica Biosystem, Nussloch, Germany).

The computed tomography (CT) method was applied using a microtomography—Skyscan 1272 (Bruker, Kontich, Belgium) and 3D visualization software—SKYSCAN 1.1.9. (Bruker, Kontich, Belgium) for the internal structure analysis. Samples with dimensions of 4 mm × 8 mm × 8 mm were scanned at 70 kV voltage, 142 µA current and 789 ms exposure with 4.5 um of voxel size. A total of 1780 and 891 sections were sliced along the two axes of the samples, respectively, and subsequently subjected to detailed evaluation.

The shrinkage of the samples was determined from the dimensional change in the transverse and longitudinal direction of 10 specimens—tensile test bars (80 mm × 10 mm × 4 mm). The measurement was performed 24 h after the injection process at a temperature of 23 °C and an air humidity of 50%.

### 2.5. Mechanical Properties

The mechanical test samples were conditioned in a chamber for 24 h at 23 °C and at 50% humidity prior to each of the mechanical testing procedures. The conditioning process during the mechanical testing was performed according to the STN EN ISO 291 standard—the temperature was 23 °C and the air humidity was 50%.

A two-column universal electromechanical testing instrument—LabTest (Labortech, Opava, Czech Republic) with pneumatic jaws and an extensometer—MFL-300B (Mess- & Feinwerktechnik, Velbert, Germany) was used for the determination of the tensile Young’s modulus, the tensile strength and the elongation at break. A 10 kN load cell was used for measurement purposes. The tensile test was performed according to ISO 527-1 2019 using 1BA test specimens with a gauge length of 25 mm. A crosshead speed of 1 mm min^−1^ was applied for the determined tensile modulus and 50 mm min^−1^ for the determined tensile strength and elongation at break. Six tensile specimens in the shape of 1BA tensile dog bones were measured for each of the two sets of tests. The tensile strength was converted from engineering values to stress-strain values for the degree of elongation of the material.

The flexural testing was performed using an dynamometer—H10KT (Tinius Olsen, Salfords, UK) according to EN ISO 178. The test specimens—tensile test bars (80 mm × 10 mm × 4 mm) were measured on a two-point platform and loaded at the third point at a speed of 2 mm min^−1^. The mean values of the flexural modulus of elasticity and flexural strength were obtained, together with deviations, following the testing of five specimens.

The impact strength was determined using the Charpy Impact method employing a pendulum impact tester—Resil 5.5 (CEAST-Instron, Italy) according to ISO 179-1/eA. The specimens—tensile test bars (80 mm × 10 mm × 4 mm) were notched with a type A notch—rN 0.25 mm. A pendulum with a nominal energy of 5 J and a 2.9 m s^−1^ striking velocity was applied. Following the measurement of 10 test specimens in the form of tensile bars (80 mm × 10 mm × 4 mm), the impact toughness was calculated and the mean value and standard deviation were calculated from the values.

The statistical comparison of the data was performed using TIBCO Statistica^TM^ software (Tibco software Inc, Palo Alto, CA, USA). The statistical significance of the mean difference of the subgroups was tested using the non-parametric Mann-Whitney test. *p*-values of 0.05 or less were considered to be statistically significant.

## 3. Results and Discussion

**Figure 2:**
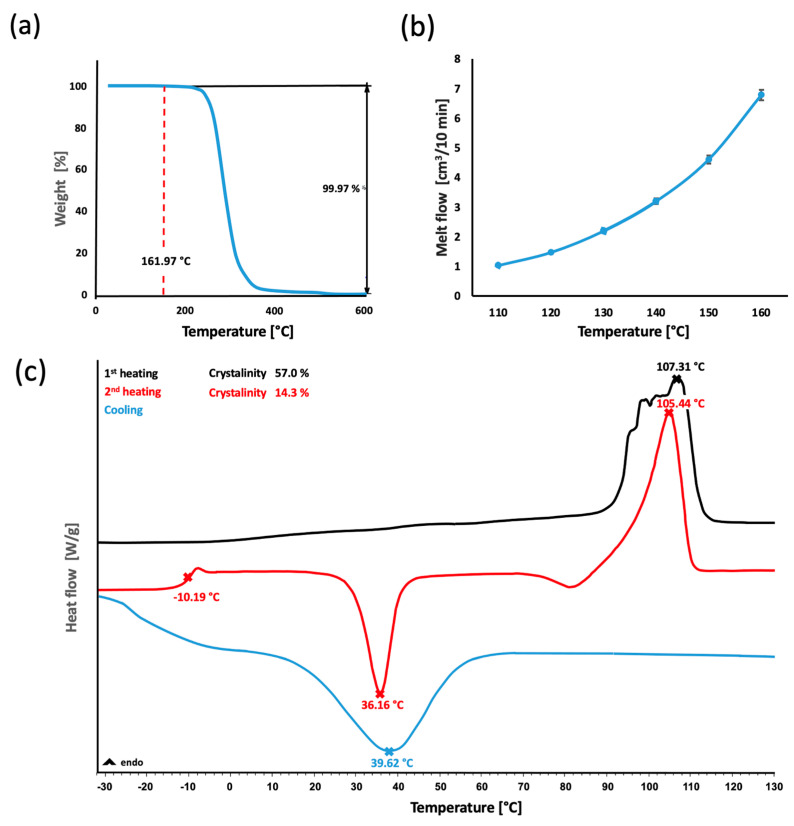
Thermal properties of the PDO granulate: TGA analysis of the thermal degradation−(**a**), MVR rheological measurement−(**b**) and DSC curves of the crystallinity and transition temperatures−(**c**).

As mentioned previously, despite the fact that the thermal and rheological properties of PDO have been well documented in the literature, it was necessary to characterize the polymer in the first stage of the experimental process (Figure 2) in the context of the standard application of the polymer in the field of the production of monofilaments employing extrusion followed by elongation. It was possible to determine the suitability of the polymer for the application of injection molding technology and to determine the scope of the experimental research via an approach that minimizes the potential degradation of the PDO material. The thermal stability temperature point was determined using TGA analysis (Figure 2a). It was still possible to thermally strain the material to a maximum temperature of 162 °C without a loss in sample weight, thus suggesting the offset of degradation. Therefore, this value represented the upper value of the technological processing “window” during the experimental process. The measured MVR values (Figure 2b) confirmed that the flow properties of PDO are suitable for the application of the considered technology. The MVR increased exponentially in the temperature range of 110–160 °C and attained values of 1.04 ± 0.01 to 6.79 ± 0.18 cm^3^/10 min. The DSC analysis (Figure 2c) identified the transition temperatures from the second heating curve following the removal of the thermal history of the granulate at values of T_g_ −10.19 °C and T_m_ 105.44 °C. The cooling curve revealed a significant zone of cold PDO crystallization in the range of 20–60 °C. This information was important in terms of the subsequent choice of the thermal profiles of the mold for injecting purposes, and it was vital that one of the profiles passes continuously through the crystallization window, thus allowing for the formation of the most homogenous crystalline phase. This also corresponds to the results in the study [27], which serves as a partial basis for this research. However, some results diverge due to the fact that the authors loaded the PDO at 200 °C, which could have led to a partial degradation of the PDO and thus the distortion of some results.

**Figure 3:**
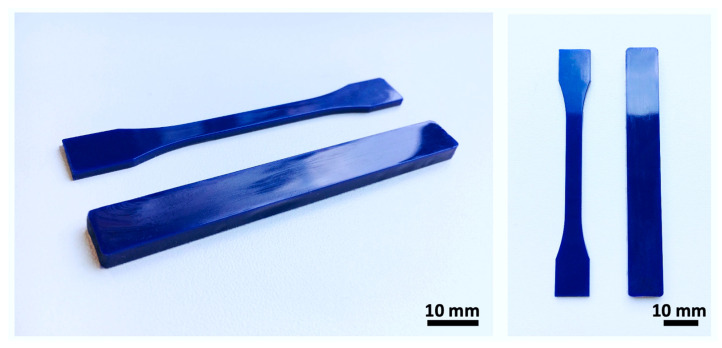
Images of the mechanical test specimens after molding process from different views.

A technological window was identified according to the thermal and rheological properties of the PDO. Based on this finding during the initial experiment, the ideal processing conditions were determined for the injection molding of the PDO (Table 1). The parameters were set in a way that applied the lowest thermal and pressure strain values, thus ensuring that the prevention of potential degradation did not occur. The use of lower values (thermal or pressure) would have led to the insufficient filling of the mold and a consequent decrease in the homogeneity of the product. The 0.4 MPa injection pressure and 0.6 MPa holding pressure are lower than commonly used values in industrial injection molding, which must be taken into account. It was mentioned previously that the two thermal states for the processing of PDO were determined based on the DSC analysis of the input polymer from the cold crystallization zone located on the cooling curve (Figure 2c). The polymer melt was injected into the mold during the first state at a temperature of 25 °C. The second state used a mold heated to 65 °C. Both states were allowed a constant 240 s cooling period, which was followed by the removal of the samples. The samples were subsequently left in a drying chamber for 24 h. The various thermal profiles ensured changes in the homogeneity and morphology of the crystalline phase, which led to a major change in the mechanical properties of the samples. It should be mentioned here that the 240 s cooling period is longer than the industrial times of around 60 s, but it was necessary to choose this time in order to ensure a demonstrable effect of different temperature effects on the final morphology. There was concern that a shorter time might disrupt this part of the experiment. During processing under the afore-mentioned conditions, the products showed a high surface quality, no undesirable deviations in terms of the shape, and no other defects (Figure 3). The sample production process progressed smoothly even without the use of the separator, which could, potentially, have left trails of chemical additives. In view of the potential application of the material in medical practice, it was deemed necessary to avoid the use of chemicals wherever possible. The shrinkage values of the samples were 1.65 ± 0.07% for 25 °C and 1.82 ± 0.15% for 65 °C. These shrinkage values corresponded to typical values for thermoplastics with a higher crystallinity value. Considering the laboratory micro-molding equipment used and the non-standard size of the samples, it was not possible to determine the shrinkage values under standardized conditions and they should be taken as indicative.

**Figure 4:**
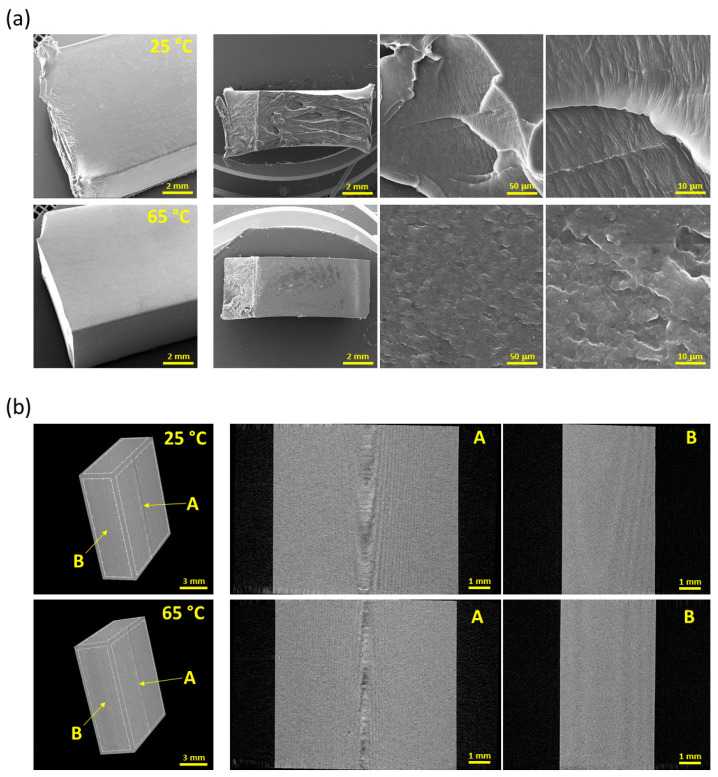
Morphology characterization imaging: SEM images showing the surface morphology of the samples on the left and the fracture morphology following the Charpy impact test at various magnifications on the right−(**a**) and CT images showing a 3D preview of a scanned sample with the scanning directions marked on the left and images showing sections of the internal structure from two sides (views from direction A and B) perpendicular to each other on the right−(**b**).

The quality of the resulting samples was determined while studying the surface and fracture morphology by means of SEM (Figure 4a) and the internal morphology via CT (Figure 4b). The surface and inner defects of the bars, such as cracks, cavities and other structural anomalies, were tested using tensile testing. Any defect would lead to the degradation of the mechanical properties. The SEM analysis of the surface revealed that both sets of samples had smooth surfaces with a minimum of inhomogeneities. Very fine structures were apparent that corresponded to the surface of the mold. SEM was also used to evaluate the fractures caused by the Charpy impact test. Images of these fractures illustrated recognizable notching zones and a surface that was created during the testing process. The edge of the fracture of the sample at 25 °C was observed to be deformed, thus indicating that the material has a relatively stiff character. The surface of the fracture was non-homogeneous and featured clear transitions and cracks that indicated the inconsistency of the structure. A higher magnification allowed for the detection of a very fine and clear lamellar structure in the form of singular lamellas with diameters of approx. 1 μm. Conversely, the cross-section of the sample at 65 °C was observed to be undeformed and sharply demarcated, thus indicating a higher level of fragility. The surface of the breakage was smooth, homogenous, and without any significant transitions, thus resulting in the high homogeneity of the structure. The higher magnification of the surface of the breakage revealed a very homogenous globular structure with grain sizes of up to 10 μm.

The CT scans of the internal structure of the samples (parts of the tensile test bars) taken in two axes served both for the evaluation of the internal structure and to provide support for the results of the evaluation of the surface quality previously conducted using SEM. The 3D scans provided by the CT method also illustrated the high quality of the surfaces of both samples. The internal structure of the samples was evaluated using CT scanning in two axes perpendicular to each other. The scans did not reveal any dark or high-contrast areas that would have indicated the presence of cavities, cracks or extraneous impurities. The images show only a small distortion in the form of artifacts in the middle section of views for direction A (Figure 4b) of the sample, which, however, did not correspond to any type of material defect.

**Figure 5:**
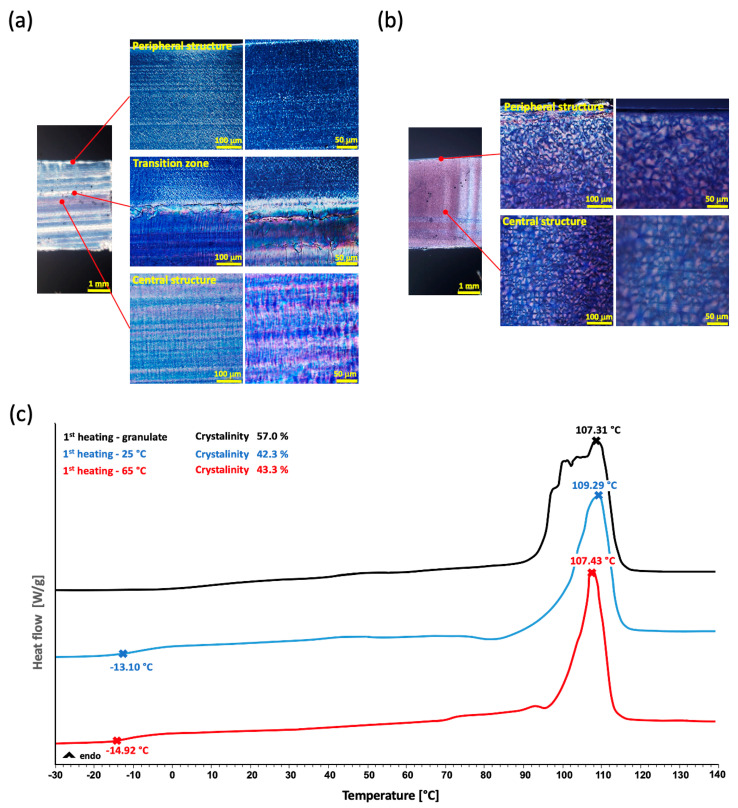
Images of the inner structure analysis: POM images of the inner structure for the 25 °C sample−(**a**), POM images of the inner structure for the 65 °C sample−(**b**) and DSC curves of the 1st heatings of both samples−(**c**).

The study of the morphology of the crystalline structure based on the temperature of the mold was conducted via the PM microscopy of thin cuts (Figure 5a,b) that were prepared in the axial direction of the flexural test bar-shaped samples, which provided a clear and flat area in contrast to the dog bone-shaped samples. Three layers were observed following the cutting of the 25 °C sample. The peripheral and central layers showed a very fine lamellar structure. The other visible layer was thin and unclear and represented a transition zone between the other two layers. During the preparation of the cuts (25 °C), the partial delamination of the layers was observed. In line with the theory, the 65 °C samples produced a uniform structure with clearly visible and larger spheroids (compared to the 25 °C samples). The formation of this crystalline structure is presumably caused by gradual cooling in the entire range of the cold crystallization zone of 20–60 °C determined by DSC (Figure 2c). This mechanism of forming the morphology of the crystalline phase structure is completely consistent with the theory in [27]. The morphology of the crystalline phase for each of the examined samples corresponded to the fractured morphology observed via SEM when evaluated using PM.

The subsequent DSC analysis demonstrated that the curves of the two samples featured no significant differences when heated for the first time (Figure 5c). The transition temperatures were similar with T_g_ values of −13.10 °C vs. −14.92 °C and T_m_ values of 109.29 °C versus 107.43 °C. This demonstrates that the differing temperatures of the mold and the subsequent cooling thermal profiles do not significantly affect the properties of the material at the molecular level. Although it was discovered that the morphology of the crystal phase for both temperatures changes significantly, the total crystalline phase values in the samples were very similar, i.e., 42.3% for 25 °C and 43.3% for 65 °C. This confirms the previous finding that the different thermal profile does not affect the value of the proportion of the crystalline phase, but only the morphology and homogeneity of the crystallites. These values are lower compared to the granulate itself, which had a crystallinity of 57.04%, which is the same value as they describe [5,8]. This is the expected difference considering that the granulate was created in the form of drawn monofilament under tension in one direction and controlled cooling, which are more ideal conditions compared to molding. In accordance with the theory, the high proportion of the crystalline phase of the granulate made it impossible to show the T_g_ region on the DSC curve.

The values of the proportion of the crystalline phase based on the second heating are very similar for both samples and for the granulate itself (Table 2). This shows that there were no changes in the chains of the molecules themselves, which would indicate a possible degradation of PDO.

**Figure 6:**
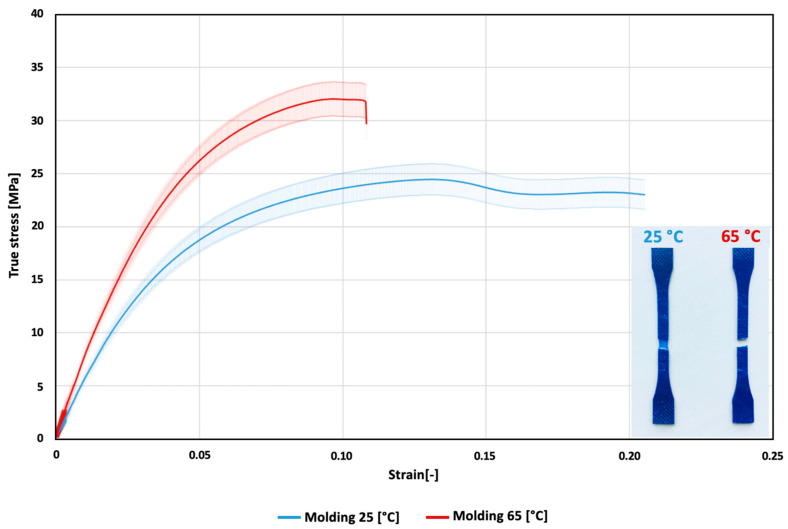
Tensile curves of the average values together with standard deviations for the 25 °C and 65 °C samples.

The stress-strain curves from the tensile tests of the PDO samples from the two injection molding scenarios reflected their completely different predicted behavior (Figure 6) caused by the emergence of a different morphology of their crystalline phase structure. The samples injected at 25 °C exhibited a lower degree of stiffness with a high-plasticity strain-softening zone, whereas the samples injected at 65 °C were stiffer and exhibited typical ductile behavior under deformation. A sudden drop is visible in the curve following the attainment of the strength and break yield point.

**Figure 7:**
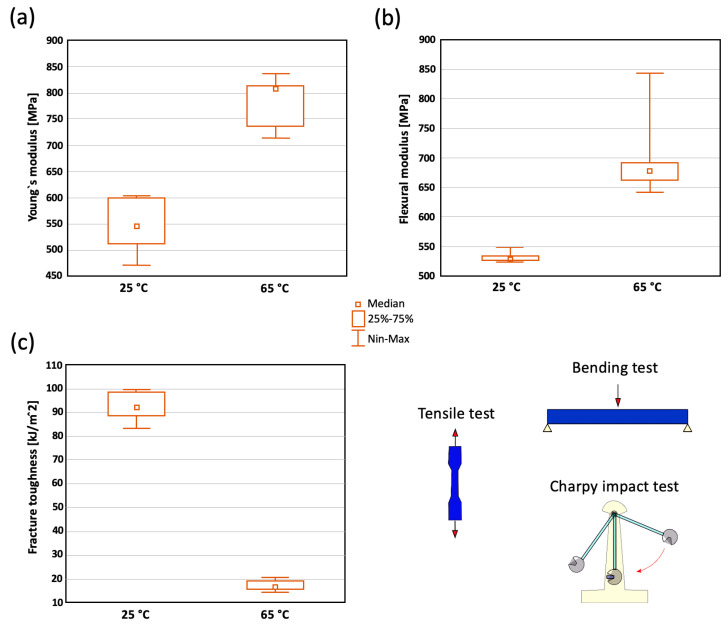
Boxplots illustrating the mechanical properties of the 25 °C and 65 °C samples; tensile modulus−(**a**), flexural modulus−(**b**) and fracture toughness−(**c**).

The average Young’s modulus observed for the samples with a molding temperature of 25 °C was 545 ± 47 MPa, whereas the average Young’s modulus observed for the samples with a molding temperature of 65 °C was 786 ± 48 MPa; the difference was statistically significant (*p* = 3.405 × 10^−3^. The same trend was observed in the flexural testing results. The average flexural modulus observed for the samples with a molding temperature of 25 °C was 533 ± 9 MPa, whereas the average flexural modulus observed for the samples with a molding temperature of 65 °C was 705 ± 72 MPa, which was also statistically significant (*p* = 1.12 × 10^−2^). Since the samples at the 25 °C molding temperature exhibited a high degree of plasticity, the most significant difference in the mechanical behavior was observed via the impact strength tests. The average fracture toughness observed for the samples with a molding temperature of 25 °C was 93 ± 5 kJ/m^2^, whereas the average fracture toughness observed for the samples with a molding temperature of 65 °C was 17 ± 2 kJ/m^2^, which, again, was statistically significant (*p* = 1.83 × 10^−4^). All values were shown in the graphs in Figure 7.

**Figure 8:**
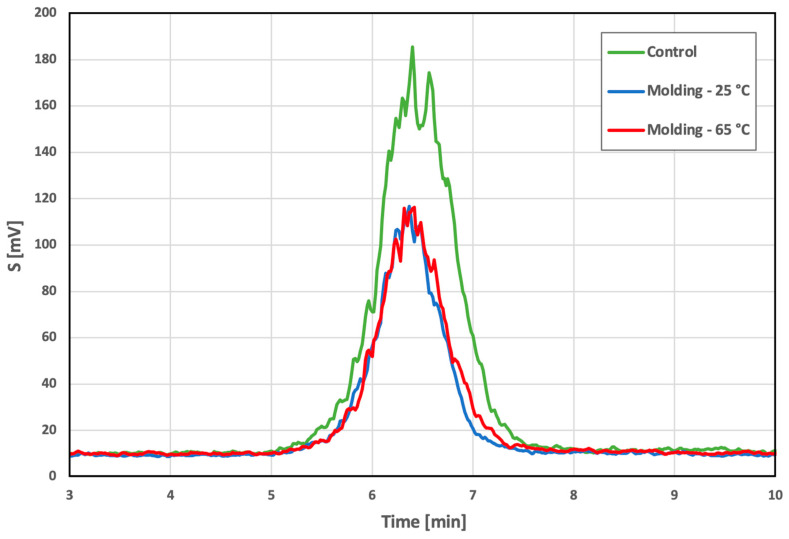
GPC chromatograms of the PDO molded samples: control (PDO granulate), molding−25 °C and molding−65 °C.

The change in the molar mass following a change in the injection molding settings was evaluated using GPS chromatography. Any change in the molar mass would potentially indicate the undesirable degradation of the material. The chromatograms in Figure 8 illustrated that the three samples exhibited identical compositions, and the data presented in Table 3 shows that the samples were evaluated at almost the same time point: 6.36 min for 25 °C, 6.41 min for 65 °C and 6.41 min for the input granulate reference. This indicated that there was no decrease in the molar mass and, thus, no degradation of the PDO during processing. In addition, this finding was confirmed by the determination of the same polydispersity values; the discrepancies in the results of this parameter were minimal and did not indicate a change in the molar mass.

Since PDO is a medical polymer and its processing employing injection molding technology is aimed particularly at the production of medical implants and for tissue engineering, cytotoxicity tests were also included in the supplementary information Figure S2. The basic test evaluated the influence of a sample extract (65 °C) placed in a cultivation medium for mice fibroblast cultivation. The test concluded that the injection molding of PDO does not result in the production of any materials with cytotoxic effects.

## 4. Conclusions

The study served to prove the potential for the processing of a biodegradable PDO human-medical polymer employing injection molding technology. The technological and process molding parameters were determined with regard to the minimization of the degradation of the material. Applying the afore-mentioned parameters, samples were produced with a very high degree of geometrical and surface fineness. Samples of the material evinced no surface or internal defects such as cavities, cracks or material impurities, a finding that was supported by both the CT and SEM analyses. The samples were molded into shapes tempered at 25 °C and 65 °C in a way that led to the occurrence of differing degrees of non-isothermal crystallization that was reflected in the differing crystalline phase morphologies, as illustrated by the SEM and PM microscopy. This difference was in the same ratio as that of the amorphous component at the two temperatures, i.e., 43.3% and 42.3%, respectively. The sample molded at a temperature of 25 °C exhibited a non-homogeneous multi-layered crystalline structure consisting of a very fine lamellar structure, whereas the samples molded at a temperature of 65 °C exhibited a highly-homogeneous crystalline structure with relatively large spheroids. The influence of the differing crystalline phase morphologies was proven via mechanical testing. The GPC analysis served to prove that the selected molding conditions did not degradation in terms of a reduction in the molar mass, and that the polymer is stable and devoid of any cytotoxic effects. Thus, the research led to the identification of a process in which the biodegradable PDO can be efficiently shaped into medical implants with the potential for significantly influencing their mechanical properties depending on the specific application requirements.

It should be noted that this work confirmed the conclusions of earlier studies describing the ability of PDO melts to crystallize into different crystalline structures upon non-isothermal cooling. However, its novelty lies mainly in proving that this mechanism is also valid for the injection molding method and very significantly affects the mechanical properties of molding parts.

The limitation of this study was the use of laboratory microinjection equipment, which did not allow for the determination of all settings and process conditions used in industrial facilities. Thus, one of the future plans is, based on the basis established in this study, to investigate the industrial scale PDO injection molding process along with all the accompanying variables. It will also be necessary to determine the degradation profile of the molded PDO.

## Figures and Tables

**Table 1:** Injection molding conditions.

Properties	Value	Unit
Melt temperature	150	°C
Mold temperature	25;65	°C
Cooling time	240	s
Injection time	3	s
Holding time	20	s
Injection pressure	0.4	MPa
Holding pressure	0.6	Mpa
Injection volume	9	cm^3^

**Table 2:** The thermal characteristic (according to DSC) of the PDO prior to (granulate) and following molding: glass transition temperature−*T*_g_, melting temperature−*T*_m_, melting enthalpy−Δ*H*_m_, and crystallinity degree−ΔΧ.

1st Heating				
Sample	*T*_g_ [°C]	*T*_m_ [°C]	Δ*H*_m_ [J/g]	ΔΧ [%]
Granulate	-	107.31	80.53	57.04
25 °C	−13.10	109.29	59.29	42.3
65 °C	−14.92	107.43	59.37	43.3
**2nd Heating**				
**Sample**	***T*_g_ [°C]**	***T*_m_ [°C]**	**Δ*H*_m_ [J/g]**	**ΔΧ [%]**
Granulate	−10.19	105.44	20.22	14.32
25 °C	−10.41	104.88	20.13	14.25
65 °C	−10.42	105.01	19.85	14.06

**Table 3:** The molar masses of the PDO prior to (granulate) and following molding: retention time−*T*_r_, number-averaged molar mass−*M*_n_, weight-averaged molar mass−*M*_w_ and polydispersity−PD.

Sample	*T*_r_ [min]	*M*_n_ [g/mol]	*M*_w_ [g/mol]	PD
Granulate	6.40	116,087	124,142	1.07
Molding—25 °C	6.36	123,571	130,737	1.06
Molding—65 °C	6.41	119,439	127,048	1.06

## Data Availability

Not applicable.

## Supplementary Materials

The following supporting information can be downloaded at: https://www.mdpi.com/article/10.3390/polym14245528/s1, Figure S1. GPC calibration curve; Figure S2. Cytotoxicity testing.
