# Supplementary material for: The Injection Molding of Biodegradable Polydioxanone—A Study of the Dependence of the Structural and Mechanical Properties on Thermal Processing Conditions
Source: Polymers (Basel). 2022 Dec 16;14(24):5528. doi: 10.3390/polym14245528 (PMC9781196; doi:10.3390/polym14245528)
Supplement: Supplementary file 1 [file polymers-14-05528-s001.zip › polymers-2069576-supplementary.pdf]

polymers

MDPI

Supplementary material

THE INJECTION MOLDING OF BIODEGRADABLE POLYDIOXANONE – a study of the dependence of the structural and mechanical properties on thermal processing conditions

Jakub Erben[1]*, Katerina Blatonova[1], Tomas Kalous[1], Lukas Capek[2], Lubos Behalek[3], Martin Boruvka[3] and Jiri Chvojka[1]

[1] Department of Nonwovens and Nanofibrous Materials, Faculty of Textile, Technical University of Liberec, 461 17 Liberec, Czech Republic; katerina.blatonova@tul.cz (K.B.); tomas.kalous@tul.cz (T.K.); jiri.chvojka@tul.cz (J.C.)
[2] Department of Technologies and Structures, Faculty of Textile, Technical University of Liberec, 461 17 Liberec, Czech Republic; lukas.capek@tul.cz (L.C.)
[3] Department of Engineering Technology, Faculty of Mechanical Engineering, Technical University of Liberec, 461 17 Liberec, Czech Republic; lubos.behalek@tul.cz (L.B.); martin.boruvka@tul.cz (M.B.)
* Correspondence: jakub.erben@tul.cz; Tel.: +420-48-535-3230

polymers

MDPI

1. Gel permeation chromatography calibration

The values of average molar masses were calculated using polymethyl methacrylate (PMMA) analytical standards (Agilent Technologies, USA) for gel permeation chromatography (GPC) analysis. The calibration curve was compiled using of five PMMA standards with different M_p values: 32,000; 54,000; 100,000; 130,000; 200,000 g/mol.

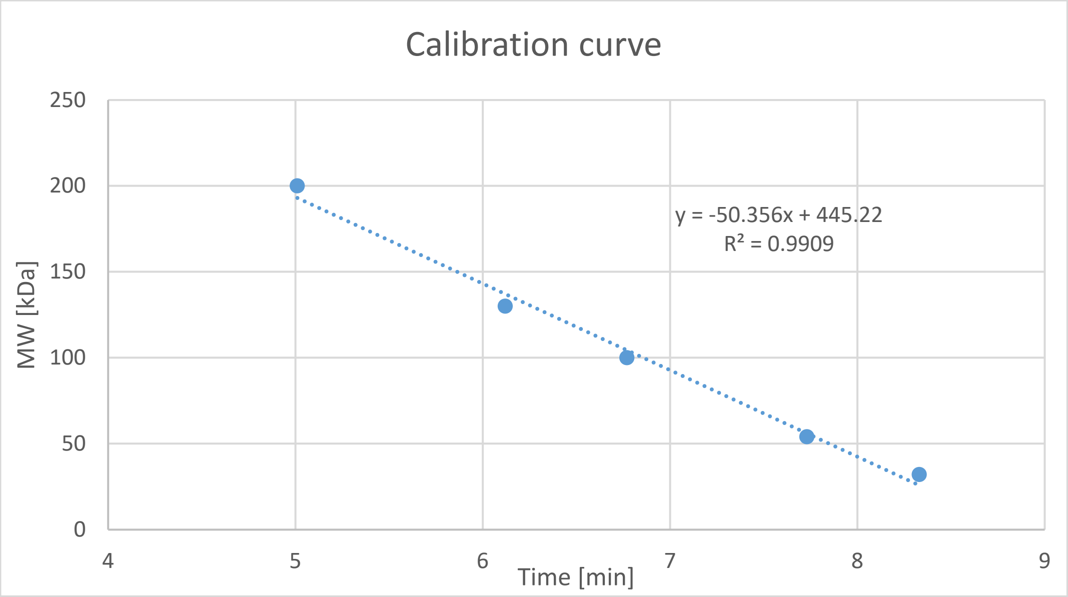

Figure S1: GPC calibration curve for PMMA analytical standards with various molecular weights.

2. Cytotoxicity testing

From the test PDO material is made an extract of the selected concentration - 10 mg / ml, 20 mg / ml. This material extract is then tested with mammalian cell culture (3T3 – mouse fibroblasts) for 24 hours in a 96 - well microtiter plate and cell viability is monitored after applying the material extract to the test cells. Viability is measured using a colorimetric assay (CCK 8, Dojindo Laboratories), which is based on the activity of enzymes (dehydrogenases) in the tested cells. Standard testing takes 72 hours. The aim is to test the possible cytotoxic effect of substances released from the tested materials.

Marking of materials and concentration of the extract:

S10 - material concentration is 10 mg / ml, number of repetitions (n) = 36

S20 - material concentration is 20 mg / ml, n = 36

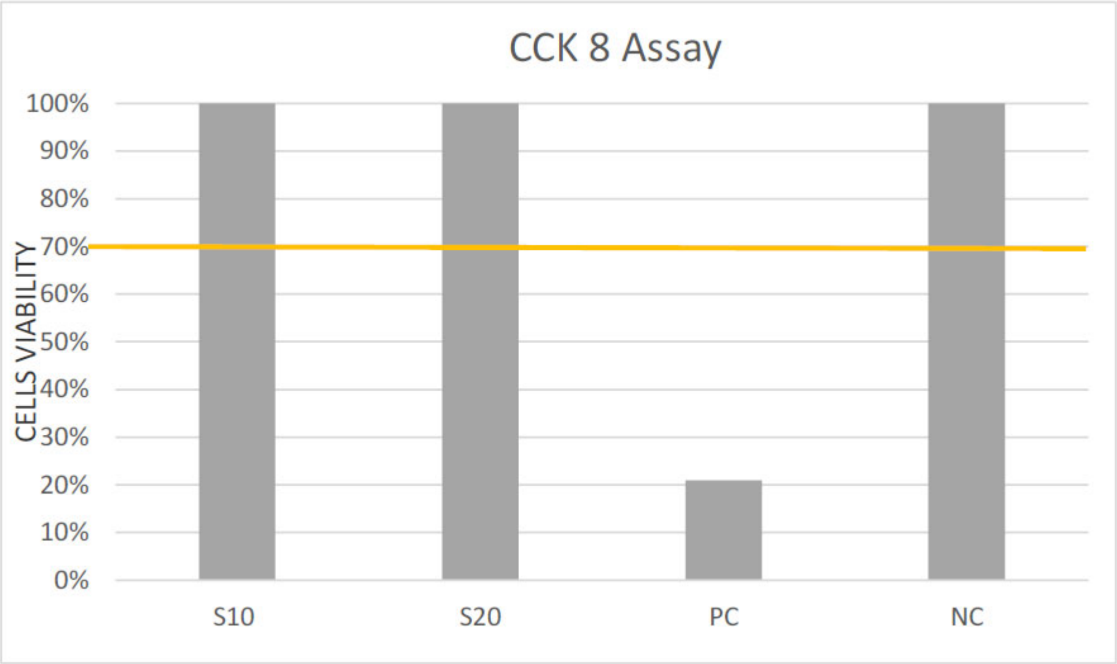

Figure S2: Cell viability (%) after 24 hours incubation with extracts from test materials (S10, S20); PC (positive control) - cytotoxic 10% triton X-100, NC (negative control) - complete medium for cell culture.
